# The chemokine receptor *cxcr5* regulates the regenerative neurogenesis response in the adult zebrafish brain

**DOI:** 10.1186/1749-8104-7-27

**Published:** 2012-07-23

**Authors:** Caghan Kizil, Stefanie Dudczig, Nikos Kyritsis, Anja Machate, Juliane Blaesche, Volker Kroehne, Michael Brand

**Affiliations:** 1 DFG-Center for Regenerative Therapies Dresden - Cluster of Excellence (CRTD), Technische Universität Dresden, Fetscherstr. 105, Dresden, 01307, Germany

**Keywords:** Adult zebrafish telencephalon, *cxcr5*, Radial glia, Proliferation, Regenerative neurogenesis, Adult neurogenesis, Differentiation

## Abstract

**Background:**

Unlike mammals, zebrafish exhibits extensive neural regeneration after injury in adult stages of its lifetime due to the neurogenic activity of the radial glial cells. However, the genes involved in the regenerative neurogenesis response of the zebrafish brain are largely unknown. Thus, understanding the underlying principles of this regeneration capacity of the zebrafish brain is an interesting research realm that may offer vast clinical ramifications.

**Results:**

In this paper, we characterized the expression pattern of *cxcr5* and analyzed the function of this gene during adult neurogenesis and regeneration of the zebrafish telencephalon. We found that *cxcr5* was upregulated transiently in the RGCs and neurons, and the expression in the immune cells such as leukocytes was negligible during both adult neurogenesis and regeneration. We observed that the transgenic misexpression of *cxcr5* in the ventricular cells using dominant negative and full-length variants of the gene resulted in altered proliferation and neurogenesis response of the RGCs. When we knocked down *cxcr5* using antisense morpholinos and cerebroventricular microinjection, we observed outcomes similar to the overexpression of the dominant negative *cxcr5* variant.

**Conclusions:**

Thus, based on our results, we propose that *cxcr5* imposes a proliferative permissiveness to the radial glial cells and is required for differentiation of the RGCs to neurons, highlighting novel roles of *cxcr5* in the nervous system of vertebrates. We therefore suggest that *cxcr5* is an important cue for ventricular cell proliferation and regenerative neurogenesis in the adult zebrafish telencephalon. Further studies on the role of *cxcr5* in mediating neuronal replenishment have the potential to produce clinical ramifications in efforts for regenerative therapeutic applications for human neurological disorders or acute injuries.

## Background

The zebrafish, unlike mammals, can regenerate its adult brain and therefore has become an excellent system to study adult neurogenesis and brain regeneration in recent years [1-6]. The adult neurogenic capacity of zebrafish is in part due to widespread stem cell niches and the neurogenic regions located in the brain [1,2,6-14]. The zebrafish has sixteen ventricular stem cell zones along the entire anterior-posterior brain axis [8]. One of these proliferation zones is located dorsally along the ventricle in the telencephalon [7,8]. The dorsal telencephalic progenitors, which show radial glial cell (RGC) morphology and are positive for glia markers such as S100β and *her4.1*, display constitutive neurogenic activity during the life-span of the fish [12,15]. In addition to the constitutive levels of adult neurogenesis, zebrafish brain can also induce a regenerative response after traumatic injury [3-6]. For instance stab wounds in the adult fish brain induce cell death, inflammation, and proliferation of RGCs [3]. Using a Cre-lox lineage tracing strategy, these cells were shown to give rise to newborn neurons, which migrate to the lesioned site and integrate into the existing brain circuitry [3]. Therefore, zebrafish offers a unique opportunity for understanding the molecular basis of adult neurogenesis and brain regeneration in vertebrates. However, the molecular programs and the genes involved in these processes are largely unknown in zebrafish.

In order to identify genes involved in adult neurogenesis and regeneration response of the adult zebrafish telencephalon, we performed an *in situ* hybridization (ISH) screen. We identified that the gene *cxcr5* was expressed in the ventricular region of the zebrafish telencephalon. *cxcr5* encodes for a G-protein-coupled chemokine receptor with seven transmembrane domains [16]. The extracellular domain at the N-terminus is critical for ligand binding whereas the intracellular part is involved in G-protein interaction and activation [16,17]. The receptor was first isolated from human Burkitt’s lymphoma and is also known as the Burkitt lymphoma receptor-1 (BLR-1) [18,19]. The ligand for *cxcr5* is the B-cell-attracting chemokine 1 (BCA-1), also known as Cxcl13 [20,21]. In mammals, *cxcr5* is expressed by mature B cells and in a subset of T cells in the immune system [22,23]. CXCR5 and CXCL13 are responsible for the organization of B cell follicles and for directing T-helper cells to the lymphoid follicles in humans [24,25]. Furthermore, the chemokine CXCL13 attracts and maintains B and T cells during inflammation [23]. Besides the immune system, cxcr5 is found in the central nervous system in adult mice in the granule and Purkinje cell layer of the cerebellum [26-28]. The receptor is also expressed in human neuronal precursor cells, which migrate across the blood vessels in the brain upon exposure to CXCL13 [29]. However, the role of *cxcr5* gene in the adult neurogenesis and the regeneration of the central nervous system in vertebrates is unknown.

In our study, we analyzed the expression pattern and the function of *cxcr5* during adult neurogenesis and regeneration of the zebrafish telencephalon. We show that *cxcr5* is detectable in the RGCs and neurons, during both adult neurogenesis and regeneration. We observed that the transgenic misexpression of *cxcr5* in the ventricular cells using dominant negative and full-length variants of the gene resulted in reduced and increased proliferation and neurogenesis response of the RGCs, respectively. When we knocked down *cxcr5* using antisense morpholinos and cerebroventricular microinjection [30], we also observed reduction of regenerative neurogenesis - an outcome similar to the overexpression of the dominant negative *cxcr5* variant using transgenic tools. We propose that *cxcr5* is an essential cue for ventricular cell proliferation and regenerative neurogenesis in the adult zebrafish telencephalon after injury.

## Results and discussion

### *cxcr5* is expressed in radial glial cells and neurons in the adult zebrafish telencephalon

**Figure 1:**
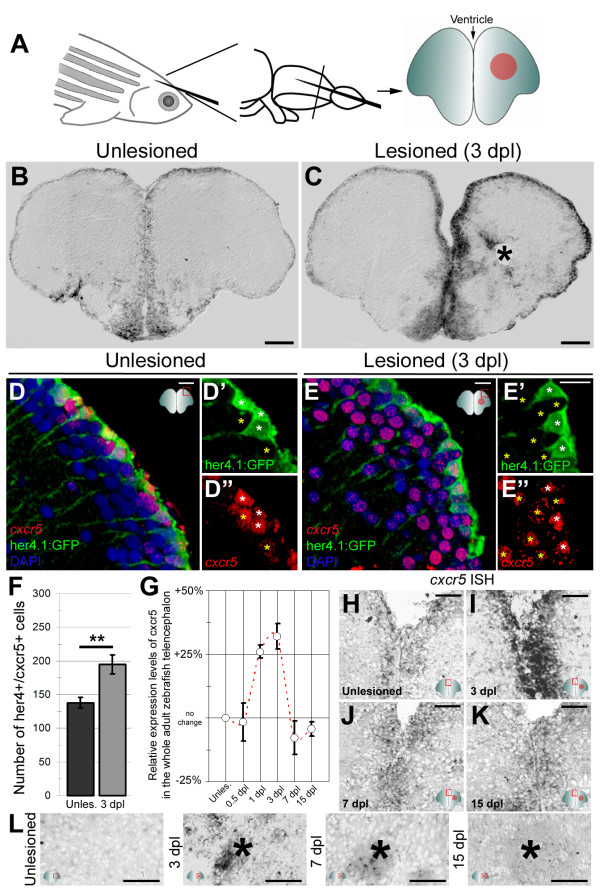
** *cxcr5* is expressed in radial glial cells (RGCs) and neurons in the adult zebrafish telencephalon. (A)** Schematic representation of an adult zebrafish telencephalon. A stab lesion is performed in one hemisphere (red circle on the cross section scheme). **(B)***cxcr5* is expressed along the ventricular region in the unlesioned telencephalon. **(C)***cxcr5* expression after a lesion (asterisk) is stronger in the lesioned hemisphere along the ventricular region. **(D)***cxcr5* fluorescent *in situ* hybridization (FISH) coupled to green fluorescent protein (GFP) immunohistochemistry in *Tg(her4.1:GFP)* transgenics in unlesioned the adult zebrafish telencephalon; counterstained with 4,6-diamidino-2-phenylindole (DAPI). **(D’)** Individual channel for her4.1:GFP. **(D”)** Individual channel for *cxcr5*. Radial glial cells (white asterisks) and periventricular cells (yellow asterisks) express *cxcr5*. **(E)***cxcr5* FISH coupled to GFP staining in *Tg(her4.1:GFP)* transgenics in the 3 day post-lesion adult zebrafish telencephalon; counterstained with DAPI. **(E’)** Individual channel for her4.1:GFP. **(E”)** Individual channel for *cxcr5*. Radial glial cells (white asterisks) and periventricular cells (yellow asterisks) express *cxcr5*. Note the number of *cxcr5*-positive periventricular cells increased in comparison to the unlesioned region. **(F)** Graph indicating the number of *her4-cxcr5* double-positive cells before and after inducing the lesion. **(G)** Quantitative real-time PCR analysis for *cxcr5* expression at different time points after the lesion. **(H-K)** Time-course *cxcr5 in situ* hybridization analyses on the unlesioned region **(H)**, 3 dpl **(I)**, 7 dpl **(J)** and 15 dpl **(K)** telencephalons. **(L)***cxcr5* expression around the lesion site. Lesion site is denoted by an asterisk; n ≥ 3 telencephalons for every analysis. Scale bars 50 μm **(B, C, H-L)**, and 10 μm **(D-E”).**

*In situ* hybridization on cross sections of the telencephalon (Figure 1A) showed that *cxcr5* is expressed in cells along the ventricular zone and close to the ventricular surface in the homeostatic state (Figure 1B). A stab lesion enhances *cxcr5* expression in the ventricular and periventricular zone, predominantly in the lesioned hemisphere (Figure 1C). We also observed *cxcr5* expression in a small number of cells close to the lesion site (Figure 1C, asterisk). These results indicate that *cxcr5* is present in the adult zebrafish telencephalon during homeostasis, and its expression is significantly enhanced at the ventricular region after traumatic injury, suggesting that *cxcr5* may be involved in adult neurogenesis and regenerative response of the telencephalon.

The expression of *cxcr5* cells along the ventricle suggests that these are progenitor cells [1,2,6]. Therefore, we analyzed whether the *cxcr5* gene is expressed in *her4.1*-positive RGCs, which act as neurogenic progenitors during adult neurogenesis and regeneration [3,12,15,31]. We performed fluorescent *in situ* hybridization (FISH) for *cxcr5* coupled to immunohistochemistry to detect the reporter activity in RGCs of the transgenic line *Tg(her4.1:GFP)*[32]. In the unlesioned (Figure 1D-D”) and lesioned (Figure 1E-E”) telencephalons, *cxcr5* is expressed in the *her4.1*-positive RGCs located along the dorsal ventricle (white asterisks) and the total number of *cxcr5*-positive radial glial cells increase significantly after inducing the lesion (Figure 1F). We also observed *cxcr5* expression in *her4.1*-negative cells (Figure 1D-E”, yellow asterisks), which are located close to the ventricle in the periventricular region, suggesting that *cxcr5* is also expressed in neurons. Based on our quantitative real-time PCR (qRT-PCR) analyses, we found that upregulated *cxcr5* expression reduces back to unlesioned levels after 3 dpl (Figure 1G), as the expression pattern of *cxcr5* at 7 dpl and 15 dpl resembles the unlesioned brains (Figure 1H-K). Additionally, the expression of *cxcr5* around the lesion site diminishes after 7 dpl (Figure 1L). These results suggest that *cxcr5* might have a transient early role during the regeneration response of the adult zebrafish telencephalon.

**Figure 2:**
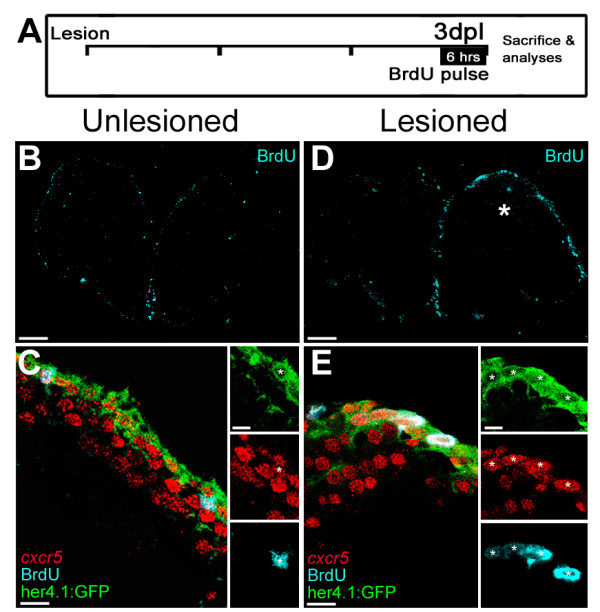
** *cxcr5* is expressed in proliferating radial glial cells (RGCs). (A)** Scheme for experimental setup. At 3 days after a lesion or a sham operation, bromo-deoxyuridine (BrdU) is applied for 6 hours before sacrificing the animals. **(B)** BrdU immunohistochemistry on the unlesioned (sham-operated) adult zebrafish telencephalon section showing the proliferating cells. **(C)***cxcr5* fluorescent in situ hybridization (FISH) coupled to BrdU and GFP immunohistochemistry on unlesioned *Tg(her4.1:GFP)* transgenics. Insets show single channel images. BrdU-positive RGCs express *cxcr5* (white asterisk). **(D)** BrdU immunohistochemistry on the 3 days post-lesion (dpl) adult zebrafish telencephalon section. Asterisk indicates the lesion site. **(E)***cxcr5* FISH coupled to BrdU and GFP immunohistochemistry on 3 dpl *Tg(her4.1:GFP)* transgenics. Insets show single channel images. BrdU-positive RGCs increase in number and they express *cxcr5* (white asterisks). Scale bars 50 μm in B and D, and 10 μm in C and E; n = 3 telencephalons.

The RGCs of the adult zebrafish telencephalon are the proliferative neurogenic progenitor cells during adult neurogenesis and regeneration [1,2,6]. Additionally, from *in situ* hybridizations and BrdU immunostainings, we observed that *cxcr5* expression overlaps with the ventricular cell proliferation (Additional file 1: Figure S1). Thus, to determine whether *cxcr5* is expressed in proliferating RGCs, we treated the *Tg(her4.1:GFP)* animals with BrdU for 6 hours before sacrifice (Figure 2A) and performed *cxcr5* FISH coupled to double immunohistochemistry for her4.1:GFP and BrdU (Figure 2B-E). In the unlesioned telencephalons, there are few proliferating cells at the ventricular region, reflecting the constitutive neurogenesis of the adult zebrafish brain (Figure 2B). We found that in unlesioned telencephalons, BrdU-positive RGCs express *cxcr5* (Figure 2C, white asterisk). After injury, the proliferating RGCs dramatically increase in number (Figure 2D), and they were also *cxcr5*-positive (Figure 2E, white asterisks). These results indicate that in both the unlesioned and the lesioned telencephalons, BrdU-labeled *her4.1*-positive RGCs express *cxcr5*, suggesting a role of *cxcr5* in proliferating radial glial cells.

ISH for *cxcr5* showed that in addition to the RGCs, *cxcr5* was also expressed close to the ventricular surface in cells that are not radial glia (Figure 1, Figure 2C, 2E). To test whether those *cxcr5*-positive cells are neurons, we used the transgenic line *Tg(HuC:GFP)*[33] in combination with the FISH for *cxcr5*. We observed *cxcr5* expression in HuC-positive neuronal cells in unlesioned (Additional file 2: Figure S2A) and lesioned telencephalons at 3 days post-lesion (dpl) (Additional file 2: Figure S2B), and a significant increase in the number of *cxcr5*-positive neurons upon lesion (Additional file 2: Figure S2C). The expression of *cxcr5* in non-glial periventricular cells (Figure 1, Figure 2) suggests that *cxcr5* is present in precursor cells that have committed to differentiate into neurons. These cells express the transcription factor NeuroD [34,35]. To determine whether the *cxcr5* is present in these cells, we performed a chromogenic *in situ* for *cxcr5* and immunohistochemistry for NeuroD (Additional file 2: Figure S2C, D). We found that *cxcr5* can be detected in the vast majority of NeuroD cells in the unlesioned and lesioned telencephalons (white asterisks in Additional file 2: Figure S2C and S2D), indicating that *cxcr5* is also expressed in differentiating neurons and thus might have a role in neuronal differentiation.

*Cxcr5* is expressed in mature B cells and in a subset of T helper memory cells in the immune system of mammals [26] and traumatic injury in the vertebrate brain induces acute inflammation that includes these cells [36-39], suggesting that *cxcr5* might be expressed in immune cells in the telencephalon. To address this, we performed ISH for *cxcr5* and L-Plastin (a marker for microglia and leukocytes, [40] and GFP immunohistochemistry on telencephlons of *Tg(her4.1:GFP)* transgenic fish before and after lesioning. In unlesioned and lesioned telencephalons, we found that L-Plastin-positive cells do not express *cxcr5* (Additional file 3: Figure S3), indicating that *cxcr5* is not expressed in leukocytes in the adult zebrafish telencephalon. These findings suggest that *cxcr5* is present only in the RGCs and the neurons in the adult zebrafish telencephalon.

### *cxcr5* regulates the proliferative capacity of RGCs and neuronal differentiation after injury

**Figure 3:**
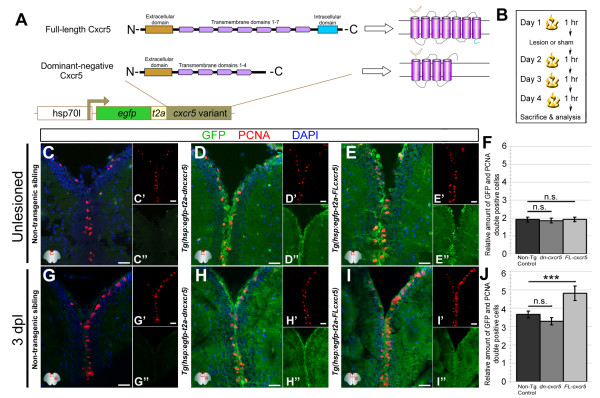
** *cxcr5* is sufficient to increase ventricular cell proliferation after injury in the adult zebrafish telencephalon. (A)** Cxcr5 is a seven-span transmembrane protein with an extracellular receptor domain, seven transmembrane domains and a C-terminus intracellular domain. We generated full-length and dominant-negative versions of Cxcr5. Both variants are inserted into a transgenesis cassette containing the enhanced green fluorescent protein (EGFP) reporter and self-cleaving T2A. The whole cassette is expressed under heat-inducible *hsp70l* promoter. **(B)** Heat shock scheme. Four heat shocks, three of which are after the lesion or sham operation, were given before sacrifice and analysis. **(C)** Proliferating cell nuclear antigen (PCNA) and green fluorescent protein (GFP) immunohistochemistry (IHC) in telencephalons of unlesioned (sham-operated) non-transgenic animals. C’ and C” are PCNA and GFP. **(D)** PCNA and GFP IHC in telencephalons of unlesioned (sham-operated) *Tg(hsp:egfp-t2a-dncxcr5)* animals. D’ and D” are PCNA and GFP. **(E)** PCNA and GFP IHC in telencephalons of unlesioned (sham-operated) *Tg(hsp:egfp-t2a-FLcxcr5)* animals. E’ and E” are PCNA and GFP. **(F)** Quantification graph for the relative amounts of GFP and PCNA double-positive cells in unlesioned telencephalons, where the transgenic misexpression of *cxcr5* does not effect cell proliferation. **(G)** PCNA and GFP IHC in telencephalons of 3 days post-lesion (dpl) non-transgenic animals. G’ and G” are PCNA and GFP. **(H)** PCNA and GFP IHC in telencephalons of 3 dpl *Tg(hsp:egfp-t2a-dncxcr5)* animals. H’ and H” are PCNA and GFP. **(I)** PCNA and GFP IHC in telencephalons of 3 dpl *Tg(hsp:egfp-t2a-FLcxcr5)* animals. I’ and I” are PCNA and GFP. **(J)** Quantification graph for the relative amounts of GFP and PCNA double-positive in 3 dpl telencephalons, where transgenic misexpression of the full-length *cxcr5* increases but the dominant negative variant does not affect the ventricular cell proliferation. Scale bars 25 μm; n = 4 telencephalons for each set of analyses.

Characterization of *cxcr5*-expressing cells indicated that *cxcr5* is expressed in proliferating RGCs and differentiating neurons of the unlesioned and lesioned adult zebrafish telencephalons (Figures 1 and 2). These results suggested that *cxcr5* may either be required for initiation of cell proliferation in RGCs (instructive function) or may be a part of a competency mechanism that imposes a proliferative potential to the RGCs (permissive function). In order to analyze the function of *cxcr5* in adult zebrafish telencephalon, we generated two transgenic lines *Tg(hsp:EGFP-T2A-dncxcr5)* and *Tg(hsp:EGFP-T2A-FL-cxcr5)*, which express a dominant-negative and a full-length variant of the gene, respectively (Figure 3A). Both of these transgenics are inducible and result in larval pigment migration phenotypes upon heat-shock (Additional file 4: Figure S4A-E). Overexpression of *cxcr5*-variants also results in alterations in the expression levels of genes known to be regulated by the *cxcr5* signaling pathway (Additional file 4: Figure S4F), suggesting that the two lines are functional. We found ubiquitous expression of the transgene in the embryos (data not shown), while the sections from the telencephalon of the heat-shocked transgenic animals (Additional file 4: Figure S4G) showed GFP-labeled cells mostly along the ventricular cells (Additional file 4: Figure S4H-J). This is either due to a possible relative silencing of the transgene in periventricular and parenchymal cells, or a positional effect of an enhancer influencing the transgene expression in the adult brain. However, the stronger expression of the transgene in the ventricular cells was advantageous for our study because it allowed us to misexpress *cxcr5* variants, particularly in the ventricular cells containing the RGCs. We also determined that the expression of the transgene after the heat-shock returns back to the levels comparable to the non-heat-shocked animals after 24 hours (Additional file 4: Figure S4K-N) suggesting that the effect of transgenic misexpression of *cxcr5* is observed only during the heat-shock period without a profound latency.

Since *cxcr5* is expressed in proliferating RGCs, we tested whether *cxcr5* has a role in proliferation of these cells in the telencephalon by using the two transgenic conditional misexpression lines: *Tg(hsp:EGFP-T2A-dncxcr5)* and *Tg(hsp:EGFP-T2A-FL-cxcr5)*. We analyzed proliferating cells in the homeostatic and regenerating telencephalons after one heat shock per day over four days (one before and three after the lesion or sham operations) (Figure 3B). In unlesioned animals, proliferating cell nuclear antigen (PCNA) and GFP immunohistochemistry (Figure 3C-E”) showed that in comparison to the non-transgenic siblings (Figure 3C), transgenic fish for both variants of *cxcr5* show no significant difference in the number of proliferating cells at the ventricle (Figure 3D-F). Following injury (Figure 3G-I), overexpression of full-length *cxcr5* significantly increased the ventricular cell proliferation (25.1 ± 8.3%; mean ± SD) (Figure 3I,J). However, overexpression of the dominant-negative *cxcr5* did not alter the number of PCNA-positive cells (Figure 3H,J). These results indicate that *cxcr5* is sufficient to promote cell proliferation of radial glial cells but may not be required, suggesting that the lack of alteration in cell proliferation in the dominant negative transgenic line might be an indication of the specificity of the effect after overexpression of the full length *cxcr5*. It is therefore possible that whether or not an RGC expressing *cxcr5* proliferates, depends not solely on *cxcr5* but also on other contextual factors (such as hypothetically, the prevalence of mitogenic factors, alleviation of suppressive mechanisms on neurogenesis, or other molecular programs activated upon the need for newborn neurons). Thus, we suggest a permissive role for *cxcr5* in RGC proliferation. This hypothesis is supported by our observations that when the full-length *cxcr5* is overexpressed, the proliferation of the RGCs increases only after injury but not during homeostasis. In such a case, even though the *cxcr5* overexpression may endow RGCs the ability to proliferate more, the prevailing signaling in unlesioned conditions is not sufficient to enhance the cell proliferation. However, after an injury, the telencephalon induces molecular programs that ultimately converge on the enhanced proliferation of the RGCs [3]. Consequently, when *cxcr5* is overexpressed following a lesion, it can increase the cell proliferation because the competent RGCs will be influenced by the injury-dependent mitogenic signals. Alternatively, the temporal dynamics of constitutive neurogenesis may be different, possibly slower, than that of the injury conditions, and this may prevent detection of significant changes in cell proliferation and neurogenesis in unlesioned brains, due to the time window we have used in our analyses. Overall, given that we observed *cxcr5* expression in proliferating RGCs and also showed that when overexpressed, *cxcr5* can enhance the proliferation of these cells context-dependently, we argue that *cxcr5* might be a permissive but not an instructive factor involved in maintaining the regenerative neurogenic capacity of the radial glial cells, by priming them for proliferation as *cxcr5* expression is sufficient but not required for proliferation of ventricular progenitors.

**Figure 4:**
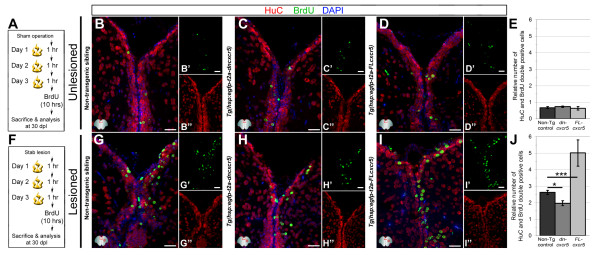
** *cxcr5* is required and sufficient for regenerative neurogenesis. (A)** Heat shock and BrdU scheme for neurogenesis assay in the unlesioned telencephalon. After a sham operation, three daily heat shocks were given before a 10-hour BrdU pulse and sacrifice at 30 days after the sham operation. **(B-D)** HuC and BrdU immunohistochemistry (IHC) and 4,6-diamidino-2-phenylindole (DAPI) counterstaining on unlesioned telencephalons from non-transgenic **(B)**, *Tg(hsp:egfp-t2a-dncxcr5)***(C)** and *Tg(hsp:egfp-t2a-FLcxcr5)***(D)** animals. Primed and double-primed images are single channels for BrdU and HuC. **(E)** Quantification graph for relative numbers of HuC and BrdU double positive cells (newborn neurons) in unlesioned telencephalons. Transgenic misexpression of *cxcr5* does not alter the constitutive levels of neurogenesis in the adult zebrafish telencephalon. **(F)** Heat shock and BrdU scheme for neurogenesis assay in lesioned telencephalons. After the lesion, three daily heat shocks were given before a 10-hour BrdU pulse and sacrifice at 30 days after lesioning. **(G-I)** HuC and BrdU IHC and DAPI counterstaining on unlesioned telencephalons from non-transgenic **(G)**, *Tg(hsp:egfp-t2a-dncxcr5)***(H)** and *Tg(hsp:egfp-t2a-FLcxcr5)***(I)** animals. Primed and double-primed images are single channels for BrdU and HuC. **(J)** Quantification graph for relative numbers of newborn neurons in lesioned telencephalons. Transgenic misexpression of dominant negative variant of *cxcr5* significantly reduces, and full-length *cxcr5* significantly increases, the number of newborn neurons after lesioning in the adult zebrafish telencephalon. Scale bars 25 μm; n = 4 telencephalons for each analysis.

We found that the *cxcr5* is expressed in differentiating neurons in addition to RGCs, which suggested a role for this gene in neurons. Thus, we hypothesized that *cxcr5* might be involved in production of neurons from the RGCs. To address this question, we performed a BrdU pulse-chase experiment using the two transgenic lines: *Tg(hsp:EGFP-T2A-dncxcr5)* and *Tg(hsp:EGFP-T2A-FL-cxcr5)* and applied four heat shocks on consecutive days, three of which were after the sham or the lesion operation. We treated the animals with BrdU at 3 dpl or 3 days post-sham (dps) and sacrificed the fish 30 days after BrdU treatment (Figure 4A). When we performed HuC (neuronal marker) and BrdU immunohistochemistry (IHC) on unlesioned non-transgenic animals (Figure 4B), *Tg(hsp:EGFP-T2A-dncxcr5)* (Figure 4C) and *Tg(hsp:EGFP-T2A-FL-cxcr5)* (Figure 4D), we found that the overall levels of neurogenesis remain unchanged upon transgenic overexpression in the unlesioned animals (Figure 4E). However, after the lesion and BrdU treatment (Figure 4F), in comparison to the non-transgenic animals (Figure 4G), overexpression of the dominant negative *cxcr5* variant significantly reduces (by 33.4 ± 5.2%, Figure 4H, J), while overexpression of full-length *cxcr5* significantly increases the number of newborn neurons (by 47.7 ± 9.4%, Figure 4I,J). These results indicate that *cxcr5* is required and sufficient for production of newborn neurons only after injury in the adult zebrafish telencephalon.

**Figure 5:**
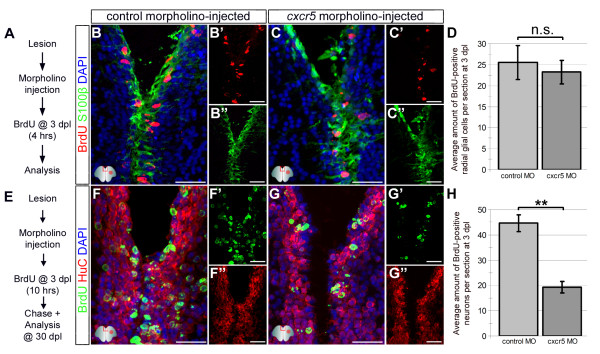
**Knocking down *cxcr5* with cerebroventricular microinjection (CVMI) results in reduced regenerative neurogenesis but not radial glial cell proliferation. (A)** Lesion, CVMI and BrdU treatment scheme. **(B)** BrdU and S100β immunohistochemistry (IHC) with 4,6-diamidino-2-phenylindole (DAPI) counterstaining on control morpholino-injected brains. **(B’)** BrdU channel. **(B”)** S100β channel. **(C)** BrdU and S100β IHC with DAPI counterstaining on *cxcr5* antisense morpholino-injected brains. **(C’)** BrdU channel. **(C”)** S100β channel. **(D)** Graph showing the average number of proliferating glial cells. Knocking down *cxcr5* does not alter the levels of proliferating radial glial cells. **(E)** Lesion, CVMI, BrdU treatment and pulse-chase scheme. **(F)** BrdU and HuC IHC with DAPI counterstaining on control morpholino-injected brains. **(F’)** BrdU channel. **(F”)** HuC channel. **(G)** BrdU and HuC IHC with DAPI counterstaining on *cxcr5* antisense morpholino-injected brains. **(G’)** BrdU channel. **(G”)** HuC channel. **(H)** Graph showing the average number of newborn neurons. Knocking down *cxcr5* significantly reduces the levels of regenerative neurogenesis.

We found that following a lesion in the adult zebrafish brain, overexpression of the dominant negative form of *cxcr5* (dn-cxcr5) does not alter ventricular cell proliferation (Figure 3J) but later reduces regenerative neurogenesis (Figure 4J). These results suggest that either the expression levels of the dn-cxcr5 was not sufficient to exert an effect on cell proliferation or *cxcr5* was not required for ventricular cell proliferation. To distinguish between these two possibilities, we took advantage of cerebroventricular microinjection (CVMI) of morpholinos [30] as an efficient tool for gene knockdown. We designed and injected *cxcr5* antisense morpholinos to the adult telencephalon after lesioning and analyzed the proliferation levels of the radial glial cells (RGC) (Figure 5A). These morpholinos are functional and the knockdown phenotypes can be rescued by *cxcr5* mRNA (Additional file 5: Figure S5). We found that the knockdown of *cxcr5* after lesion did not alter the levels of RGC proliferation (Figure 5B-D), which was consistent with the outcome of the overexpression of dn-cxcr5 (Figure 3J). When we analyzed the neurogenic response after lesioning and cerebroventricular microinjection (CVMI) of morpholinos (Figure 5E), we observed that the knockdown of *cxcr5* reduced the regenerative neurogenesis significantly (55.3 ± 7.1%, Figure 5F-H), confirming the results of overexpression of dn-cxcr5 (Figure 4J). Additionally, we found that misexpression of *cxcr5* variants did not affect the cell survival before and after lesioning (Additional file 6: Figure S6), hence, the reduction in the regenerative neurogenesis response is not due to cell death. These findings indicate that *cxcr5* is not required for ventricular cell proliferation but is required for the regenerative neurogenesis in the adult zebrafish telencephalon.

We also detected *cxcr5* in non-proliferating RGCs (Figure 3). This suggests that either the expression of *cxcr5* in non-proliferating glia reflects a physiological stage of the RGC at which it is competent to proliferate but is not actually in the cell cycle, or *cxcr5* has a proliferation-independent role in those glial cells. In adult brains, glial cells are known to be heterogeneous in terms of their proliferation and progenitor characteristics [1,2,6,12,15]. Therefore, *cxcr5* may either be expressed in glial cells with different inherent propensities to become neurogenic and proliferative or it can be a part of the mechanism that establishes the functional heterogeneity. It is not clear whether the constitutive neurogenesis and the injury-induced neurogenesis use the same progenitor cells to form neurons by simply enhancing the rate of cell divisions, or whether there are reserve stem cells that are activated after injury. Given that we observed an effect of *cxcr5* misexpression on proliferation and neurogenesis only after the injury, it is possible that the roles of *cxcr5* we identified are associated with the regenerative response. This suggests that the expression of *cxcr5* in unlesioned brains may either be relevant to a permissive proliferative and neurogenic capacity of the RGCs or be a physiological function independent of RGC proliferation. However, it is currently not possible to distinguish between these alternatives with our current level of understanding. Long-term single-cell lineage tracing experiments with a *cxcr5*-positive non-proliferative RGC will help us understand whether under homeostatic conditions, *cxcr5*-positive glial cells initiate cell division and form neurons, or whether they are required for an as-yet unidentified role in the glial cells.

**Figure 6:**
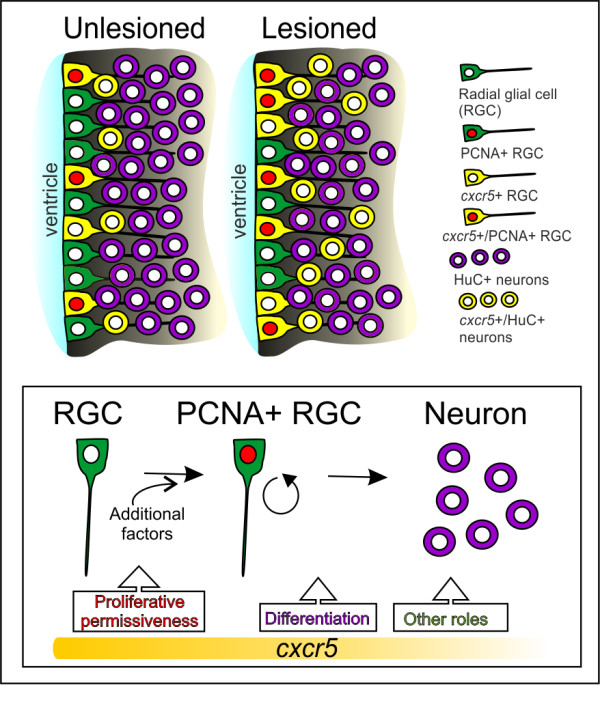
**Summary and working model for *cxcr5* expression and function in unlesioned and lesioned adult zebrafish telencephalons.** In unlesioned adult zebrafish telencephalons, *cxcr5* is expressed in proliferating and non-proliferating radial glial cells (RGCs), as well as neurons in the periventricular region. After a lesion, proliferating *cxcr5*-positive RGCs and *cxcr5*-positive periventricular neurons increase in number. *cxcr5* expression is strongest at the ventricular surface and weakens towards the deeper layers of parenchyma. Based on the transgenic misexpression of the dominant negative and the full-length variants of *cxcr5* gene in the ventricular zone cells, we propose that *cxcr5* is a permissive cue for proliferative competency of the RGCs. *cxcr5* is sufficient to enhance proliferation of RGCs not before but after injury, possibly due to additional injury-induced factors required in combination with *cxcr5* to enhance cell proliferation. Based on the expression of *cxcr5* in differentiating neurons and long-term neurogenesis experiments upon transgenic misexpression, we suggest that *cxcr5* is also required for differentiation of RGCs to neurons. Since *cxcr5* is also expressed in neurons, it might have other roles in neurons, such as migration, which are yet to be identified.

The dominant negative variant of *cxcr5* did not affect cell proliferation in unlesioned or lesioned telencephalons. Similarly, *Tg(hsp:EGFP-T2A-dncxcr5)* animals did not alter their neurogenesis levels after heat shocks in unlesioned conditions. However, when overexpressed, this variant reduced the production of neurons after the lesion. These findings pose four possible explanations. First, *cxcr5* may not be required for cell proliferation but may be required for differentiation of RGCs to neurons. Second, the different effects of dominant-negative *cxcr5* could be due to the expression levels of the *Tg(hsp:EGFP-T2A-dncxcr5)* transgenic cassette. The dose of the transgene expression might not be sufficient to compete with the endogenous levels of *cxcr5*, which could be adequate to fulfil the cognate function in the cells and obscure any loss-of-function effect. However, we believe that this option is unlikely as by injecting *cxcr5* morpholinos using CVMI to the adult fish brain, we ruled out the dosage effect. Third, *cxcr5* may have a redundant function in RGC proliferation and therefore loss of *cxcr5* function can be compensated. Finally, the transgene may not be expressed in all *cxcr5*-expressing cells. This situation would cause limitations if *cxcr5* had a paracrine effect on other cells. Thus, inability to target all *cxcr5* cells would cause a hypomorphic effect, which might hinder an overt phenotype. Identifying other factors relevant to radial glial cell proliferation and neurogenesis in adult fish brain and transgenic misexpression studies using endogenous *cxcr5* promoter may be helpful in the future to address such reservations. Nevertheless, our results currently indicate that *cxcr5* is functionally involved in regulation of progenitor cell proliferation and neuronal differentiation only after injury in the adult zebrafish telencephalon (Figure 6).

## Conclusions

We demonstrated that *cxcr5* is expressed at different physiological states of glial cells - during quiescence, proliferation and differentiation into neurons. We also demonstrated that *cxcr5* function is biologically relevant to the proliferation of the neurogenic progenitor cells and the regenerative neurogenesis response of the adult zebrafish brain (Figure 6). Therefore, we present *cxcr5* as an intriguing factor relevant to the proliferative status and differentiation capacity of the RGCs after lesion in the adult zebrafish telencephalon. In mammals, after traumatic lesions in the central nervous system, glial cells initiate proliferation but cannot replenish the lost neurons [37,41-47]. One of the detrimental cues for neurogenesis is believed to be the acute inflammation [38,48-50]. Given that zebrafish can regenerate traumatic injuries in the brain and the fish RGCs expresses *cxcr5*, which we showed to be relevant to RGC proliferation and neuronal differentiation, we believe that *cxcr5* can be an interesting candidate that may be involved in the permissive link between the immune and the nervous systems in zebrafish. Further studies on the role of *cxcr5* in mediating neuronal replenishment have the potential to produce clinical ramifications in efforts for regenerative therapeutic applications for human neurological disorders or acute injuries.

## Methods

### Ethics statement

All animal experiments were carried out in accordance with the recommendations and permits of the Landesdirektion Dresden (Permit numbers: AZ 24D-9168.11-1/2008-2, 4 and 14). All surgery was performed under anesthesia, and all efforts were made to minimize suffering. Fish were raised and kept at 28°C under a 14-hour light, 10 hours dark cycle, and fed with brine shrimp artemia daily as described [51]. Wildtype experimental animals were adult fish from the *gol-b1* line with AB genetic background [52].

### Tissue preparation and cryosectioning

Fish were sacrificed with 1% 3-amino-benzoic acid ethyl ester (MESAB, Sigma, Schnelldorf, Germany) and the skull was opened at the hindbrain area with sharp forceps to enable a better fixation of the tissue. The skull was partly removed without damaging or relocating the telencephalon. The head was cut off behind the pectoral fins with a scalpel and the brains were fixed in 2% paraformaldehyde (PFA, Sigma, Schnelldorf, Germany) at 4°C overnight. Heads were washed twice with 0.1 M phosphate buffer, transferred in 20% sucrose-ethylene-diamine-tetra-acetic acid (EDTA, Sigma, Schnelldorf, Germany) solution (in 0.1 M phosphate buffer, pH: 7.5) and incubated overnight at 4°C. Brains were then frozen in 7.5% gelatin/20% sucrose in 0.1 M phosphate buffer and cryosectioned at 14-μm thickness using a cryostat. Heads were sectioned for the telencephalon between olfactory bulb and diencephalon with sequential distribution to three slides, therefore one slide contained every third section. Sections were stored at −20°C.

### In situ hybridization, immunohistochemistry, terminal deoxynucleotidyl transferase dUTP nick end labeling (TUNEL) staining

A fragment of the zebrafish *cxcr5* gene [NCBI Accession ID: XM_003200482; Ensembl Accession ID: ENSDARG00000010514] was isolated from 2 days post-fertilization (dpf) embryonic total cDNA using the following primers: forward primer: 5′-TACATCTGGCAGTGGCTGAC-3′ and reverse primer: 5′-CAGCAGGTCTCTTCGGAAAC-3′. The amplicon (726 bp) was subcloned into pGEM T-Easy (Promega). A DIG or fluorescein-labeled mRNA probe was generated using an in vitro transcription kit (Roche, Mannheim, Germany). In situ hybridization was performed as described [12,53]. To combine *ISH* with IHC, the primary antibody for the immunohistochemistry was added with the anti-DIG or anti-Fluorescein antibody. After the in situ staining reaction, the secondary antibody was applied. IHC was performed as described [3,8,11,12,30]. Antigen retrieval was performed with Tris–HCl buffer, pH: 8.0 at 100°C for 5 minutes, 10 mM sodium citrate buffer, pH: 6.0 at 85°C for 6 minutes or 2 M HCl for 20 minutes at 37°C. The following antibodies were used: primary antibodies: anti-BrdU (rat IgG_2a_, Serotec, Düsseldorf, Germany, 1:500), anti-GFP (rabbit, Molecular Probes, Darmstadt, Germany, 1:1,000); anti-GFP (chicken, Abcam, Cambridge, UK, 1:3,000); anti-HuC/D (mouse IgG_2b_, Molecular Probes, 1:50), anti-neuroD (mouse IgG_2a_, Abcam, 1:750), Anti-Lcp1 (rabbit, 1:10,000, gift from Michael Redd, University of Utah, USA), anti-PCNA (mouse IgG_2a_, Dako Cyto, Hamburg, Germany, 1:500), anti-DIG (sheep, Roche, Mannheim, Germany, 1:4,000), anti-fluorescein (sheep, Roche, 1:5,000); secondary antibodies: goat anti-rat, goat anti-rabbit, goat anti-mouse (IgG, IgG_2a_, IgG_2b_), goat anti-chicken (coupled to Alexa-488, Alexa-555; Alexa-633, Molecular Probes, 1:500). TUNEL staining was performed using ApopTag® Red in situ apoptosis detection kit (Millipore, Darmstadt, Germany) according to the manufacturer’s instructions.

### BrdU labeling

To label cells in S-phase of the cell cycle, zebrafish were immersed in 10 mM BrdU (Sigma) solution [8]. BrdU was dissolved in E3 medium, the zebrafish were incubated for desired periods indicated throughout the text.

### Brain lesions and cerebroventricular microinjection (CVMI)

Fish were anesthetized using MESAB (0.01%). A 30-gauge cannula (Becton Dickinson Biosciences, Heidelberg, Germany), which corresponds to an outer diameter of 200 μm, was inserted through the fish nostril and stabbed along the rostro-caudal axis through the olfactory bulb until reaching the caudal part of the telencephalon as described [3]. CVMI was performed as described [30], fish were anesthetized using MESAB (0.01%). With the help of a fine needle (Becton Dickinson Biosciences) an incision was made through the skull to the cerebroventricular fluid (CVF) of the adult zebrafish above the optic tectum. This incision generates a slit through the skull with a diameter of approximately 200 μm without damaging the optic tectum underneath. Injected liquid disperses through the CVF rostrally to the forebrain. We generated con-trol (5′-CCTCTTACCTCAGTTACAATTTATA-3′) and translation-blocking antisense (5′-AACCCCTCTCCTCAAGGACTGGCAT-3′) morpholinos for *cxcr5*, tested these morpholinos in embryos for functionality and specificity (Additional file 5: Figure S5), and used them for CVMI into adult zebrafish brain.

### qRT-PCR

Quantitative RT-PCR analyses were performed as described [53] using the primers listed in Additional file 7: Table S1.

### Generation of conditional transgenic lines for *cxcr5* gene variants

For full-length *cxcr5* transgenics, complete open reading frame of the zebrafish *cxcr5* gene [NCBI accession: XM_003200482, Cxcr5: 402 aminoacids] was used (Figure 5A). For the dominant negative variant, the 5^th^, 6^th^ and the 7^th^ transmembrane domains and the intracellular C-terminal domain were deleted (Δ^243-402^-Cxcr5 (dnCxcr5), 243 aminoacids) (Figure 5A). The transgenesis cassette contains two flanking Tol2 elements [54,55], and in between these are the heat-inducible zebrafish *hsp70* promoter, the EGFP-T2A-dnCxcr5 or EGFP-T2A-FL-Cxcr5 gene sequence (See Figure 5). To integrate the construct into the zebrafish genome, transgenesis constructs were co-injected with Tol2 transposase mRNA to the one-cell stage of the embryos [54-57]. Founder animals were outcrossed to wild type strains and the progeny was screened for GFP fluorescence after single heat shock at early gastrulation stages. The fish carrying the transgenes heterozygously were outcrossed for two generations to segregate multiple insertions. The nomenclature for the transgenic animals are as follows: *Tg(hsp:EGFP-T2A-dncxcr5)* for the dominant-negative variant, and *Tg(hsp:EGFP-T2A-FL-Cxcr5)* for the full-length variant. We also used *Tg(her4.1:GFP)*[32] and *Tg(HuC:GFP)*[33] transgenic lines, which were kindly provided by the relevant laboratories.

### Heat shock paradigm

Heat-shock experiments were performed on embryos and adults. Embryos (at least 50% epiboly stage) were transferred into 2 ml tubes. After removal of the excess embryo medium (E3, 5 mM NaCl, 0.17 mM KCl, 0.33 mM CaCl_2_.2H_2_O, 0.33 mM MgSO_4_.7H_2_O, 0.0002% methylene blue, pH: 6.5), E3 preheated to 39°C was added in the tubes, which were incubated for 30 minutes at 38°C. After the heat shock, embryos were returned to Petri dishes filled with E3 and placed in a 28°C incubator. The embryos were analyzed with a fluorescent microscope at least 4 hours after the heat induction. Adult fish were transferred into the heat shock aquarium and received in total, four heat shocks on four consecutive days. Each day the water was heated to 37°C, then the heating elements switched off and the water passively cooled down to 28°C (this takes approximately 5 to 6 hours after the heat shock). The fish were analyzed with an Olympus MVX10 fluorescent microscope after anesthetization with MESAB (0.01%). Embryos were sorted and used for desired purposes.

### Imaging, quantification and statistical analysis

Fluorescence images were taken using a structured illumination microscope (Zeiss Apotome AxioImager.Z1) or a laser scanning confocal microscope (Zeiss 510 META). Quantifications were performed in an unbiased manner by counting the absolute number of cells in a minimum of eight different sections of the brain of every fish. For simplicity, results are depicted as the relative amount of cells. For statistical analysis, two-way analysis of variance (ANOVA) and Tukey’s post hoc test or paired t-test were used after determining whether the sample datasets conform to a normal distribution. P-values are indicated as follows: *P ≤ 0.05, **P ≤ 0.005, ***P ≤ 0.001. The number of fish used for every experiment is indicated in the corresponding figure legends.

## Abbreviations

ANOVA, analysis of variance; BCA-1, B-cell-attracting chemokine 1; BLR-1, Burkitt lymphoma receptor-1; BrdU, bromo-deoxyuridine; CVMI, cerebroventricular microinjection; cxcr5, chemokine (C-X-C motif) receptor 5; DAPI, 4,6-diamidino-2-phenylindole; dpf, days post-fertilization; dn, dominant negative; dpl, days post-lesion; dps, days post-sham; EDTA, ethylene-diamine-tetra-acetic acid; fl, full-length; EGFP, enhanced green fluorescent protein; FISH, fluorescent in situ hybridization; GFP, green fluorescent protein; her4.1, hairy-related 4.1 basic helix-loop-helix transcription factor; IHC, immunohistochemistry; ISH, in situ hybridization; MESAB, 3-amino-benzoic acid ethyl ester; PCNA, proliferating cell nuclear antigen; PFA, paraformaldehyde; qRT-PCR, quantitative real-time polymerase chain reaction; RGC, radial glial cell; TUNEL, terminal deoxynucleotidyl transferase dUTP nick end labeling.

## Competing of interests

The authors declare that there is no competing of interest.

## Authors’ contributions

CK conceived and designed the experiments; CK, SD and AM generated the transgenic animals; CK, SD, NK, AM, JB carried out the experiments; VK contributed to the initial screen; CK wrote the manuscript; CK and MB edited the manuscript. All authors read and approved the final manuscript.

## Supplementary Material

Additional file 1 **Figure S1.** Title: cxcr5 expression is overlapping to the proliferating ventricular cells in adult zebrafish telencephalon. (A) Lesion and bromo-deoxyuridine (BrdU) treatment paradigm. Fish were treated with BrdU 3 days post lesion (dpl) for 6 hours before the sacrifice. (B) cxcr5 in situ hybridization on lesioned telencephalon. (B**’**) High magnification of the dorsomedial region in (B). (B”) High-magnification of the dorsolateral region in (B). (C) BrdU immunohistochemistry on lesioned telencephalon. (C**’**) High-magnification of the dorsomedial region in (C). (C”) High-magnification of the dorsolateral region in (C). (D) Merged image of (B) and (C). (D**’**) Merged image of (B**’**) and (C**’**). (D”) Merged image of (B”) and (C”). Click here for file

Additional file 2 **Figure S2.** Title: Neurons express *cxcr5*. (A) *cxcr5* fluorescent in situ hybridization (FISH) coupled to HuC immunohistochemistry (IHC) on a section of unlesioned adult zebrafish telencephalon. Inset is magnified image. **(B)***cxcr5* FISH coupled to HuC IHC on a section of 3 days post lesion (dpl) adult zebrafish telencephalon. Inset is magnified image. *cxcr5* is expressed in neurons before and after injury. **(C)** Quantification graph for *cxcr5*-expressing HuC-positive cells. **(D)***cxcr5* chromogenic in situ hybridization (ISH) coupled to NeuroD IHC in unlesioned telencephalon. NeuroD-positive differentiating neurons, which are several cell diameters away from the ventricle express *cxcr5* (white asterisks). *cxcr5* expression is weaker in NeuroD-positive neurons in comparison to *cxcr5*-positive cells closer to the ventricle (yellow asterisks). In a transition zone between strong NeuroD-positive (white asterisks) and NeuroD-negative cells (yellow asterisks), cells express NeuroD and *cxcr5* weakly (blue asterisks). **(E)***cxcr5* chromogenic ISH coupled to NeuroD IHC in 3 dpl telencephalon. NeuroD-positive differentiating neurons are more numerous, dispersed inside the parenchyma distantly in comparison to unlesioned telencephalons and express *cxcr5* (white asterisks). *cxcr5* expression is weaker in NeuroD-positive neurons in comparison to *cxcr5*-positive cells closer to the ventricle (yellow asterisks). **(F)** Quantification graph for cxcr5-positive NeuroD-expressing cells. The number of NeuroD/cxcr5 double-positive cells increase upon injury in adult zebrafish telencephalon. Scale bars 25 μm; n = 4 telencephalons for every set of analyses. Click here for file

Additional file 3 **Figure S3.** Title: cxcr5 is not expressed in L-Plastin-positive cells in the adult zebrafish telencephalon. (A) cxcr5 in situ hybridization coupled to immunohistochemistry for L-Plastin (red, marking macrophages and microglia) and her4.1:green fluorescent protein (GFP) (green, marking the radial glial cells) in the unlesioned telencephalons. White asterisks indicate the L-Plastin cells. (A1-A3) Individual channels for L-Plastin, her4.1:GFP and cxcr5, respectively. (B) cxcr5 in situ hybridization coupled to immunohistochemistry for L-Plastin and her4.1:GFP in the lesioned telencephalons (dorsolateral region). White asterisks indicate the L-Plastin cells. (B1-B3) Individual channels for L-Plastin, her4.1:GFP and cxcr5, respectively. (C) cxcr5 in situ hybridization coupled to immunohistochemistry for L-Plastin and her4.1:GFP in the lesioned telencephalons (dorsomedial region). White asterisks indicate the L-Plastin cells. (C1-C3) Individual channels for L-Plastin, her4.1:GFP and cxcr5, respectively. Click here for file

Additional file 4 **Figure S4.** Title: Larval phenotypes upon *cxcr5* misexpression; and transgene activity in the adult zebrafish telencephalon. **(A)** Cxcr5 is a seven-span transmembrane protein with an extracellular receptor domain, seven transmembrane domains and a C-terminus intracellular domain. We generated full-length and dominant-negative versions of Cxcr5. Dominant negative variant lacks the last three transmembrane domains and the intracellular domain. Both variants are inserted into a transgenesis cassette that contains the coding sequence for enhanced green fluorescent protein (EGFP) and self-cleaving T2A peptide. The whole cassette is expressed under heat-inducible *hsp70l* promoter. **(B)** Non-transgenic sibling at 3 days post-fertilization (dpf). **(B1)** High-magnification image of the yolk-sac in B. **(B2)** High-magnification image of the yolk-tube extension in B. **(B3)** High-magnification image of the ventral fin fold in B. **(C)***Tg(hsp:egfp-T2A-dncxcr5)* transgenic animals at 3 dpf after two heat shocks in gastrula. **(C1)** High-magnification image of the yolk-sac in C. **(C2)** High-magnification image of the yolk-tube extension in C. **(C3)** High-magnification image of the ventral fin fold in C. **(D)***Tg(hsp:egfp-T2A-FLcxcr5)* transgenic animals at 3 dpf after two heat shocks in gastrula. **(D1)** High-magnification image of the yolk-sac in D. **(D2)** High-magnification image of the yolk-tube extension in D. **(D3)** High-magnification image of the ventral fin fold in D. **(E)** Quantification graph for the number of pigment cells in different regions of the non-transgenic, *Tg(hsp:egfp-T2A-dncxcr5)* and *Tg(hsp:egfp-T2A-FLcxcr5)* larvae. Note that dominant negative variant of *cxcr5* significantly reduces while full-length *cxcr5* significantly increases the number of pigment cells in comparison to the non-transgenic siblings. **(F)** Quantitative real-time PCR analyses at 3 dpf after the misexpression of *cxcr5* with two heat shocks at gastrula. **(G)** Heat-shock scheme for adult zebrafish telencephalon expression. **(H)** GFP and DAPI staining in heat-shocked non-transgenic animals. **(I)** GFP and DAPI staining in heat-shocked *Tg(hsp:egfp-T2A-dncxcr5)* animals. **(J)** GFP and DAPI staining in heat-shocked *Tg(hsp:egfp-T2A-FLcxcr5)* animals. **(K-M)** GFP IHC on *Tg(hsp:egfp).***(K)** Olfactory bulb at 1 day after heat-shock. **(L)** Olfactory bulb at 2 days after heat-shock. **(M)** Telencephalon at 1 day after heat-shock. **(N)** Telencephalon at 2 days after heat-shock. GFP protein as a result of heat shock paradigm reduces significantly after 24 hours after heat shock. Click here for file

Additional file 5 **Figure S5.** Title: *cxcr5* translation-blocking antisense morpholino is functional. **(A)** Uninjected 2 day post-fertilization (dpf) embryos. **(B)** Control morpholino-injected embryos show no morphological phenotypes at 2 dpf. **(C)***cxcr5* antisense morpholinoinjected embryos display severe anomalies in axial extension and head development. **(D)***cxcr5* mRNA rescues the knockdown phenotypes when co-injected with antisense morpholinos. **(E)** Quantitative real-time PCR analysis of genes regulated by *cxcr5,* upon control and antisense morpholino injections. All the genes tested are upregulated after knocking down *cxcr5*. Percentages in A-D represent the ratio of embryos with gross morphological defects to the whole clutch size. Click here for file

Additional file 6 **Figure S6.** Title: Misexpression of *cxcr5* does not lead to cell death. (A-I) TUNEL staining to detect the apoptotic cells in non-transgenic siblings, *Tg(hsp:egfpt2a-FLcxcr5)* and *Tg(hsp:egfp-t2a-dncxcr5)* animals pre-lesion, 3 days post-lesion (dpl) and 15 dpl time points. Red nuclei indicate the apoptotic cells. **(J)** Quantification graph shows the number of apoptotic nuclei in the lesioned hemisphere. Misexpression of *cxcr5* does not alter the levels of cell death. Two telencephalons were used for every time point. Click here for file

Additional file 7 **Table S1.** Title: qRT-PCR primers. Click here for file
