# Supplementary figures and images for: The chemokine receptor cxcr5 regulates the regenerative neurogenesis response in the adult zebrafish brain
Source: Neural Dev. 2012 Jul 23;7:27. doi: 10.1186/1749-8104-7-27 (PMC3441421; doi:10.1186/1749-8104-7-27)

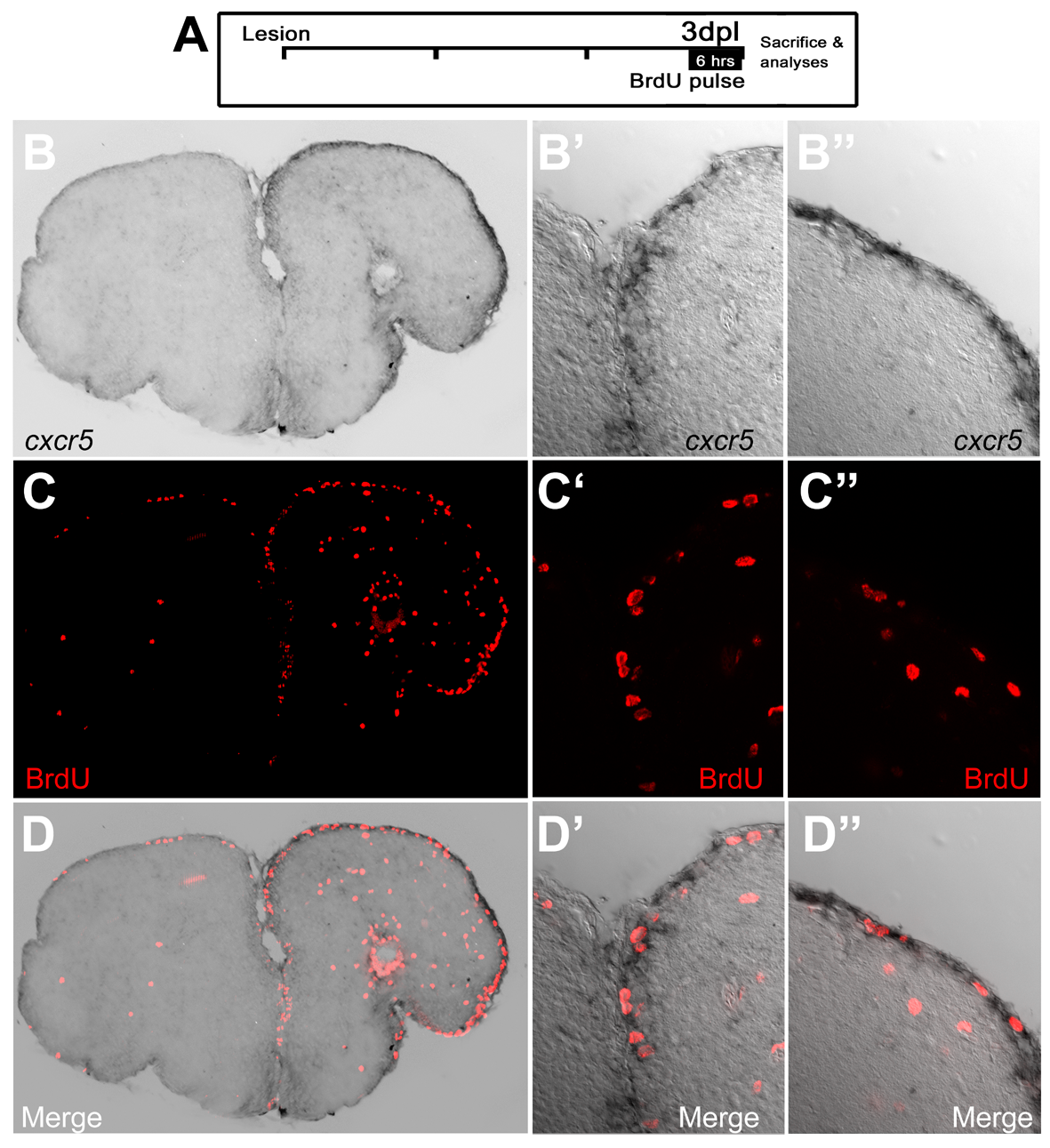

Supplement: Additional file 1 — Figure S1. Title: cxcr5 expression is overlapping to the proliferating ventricular cells in adult zebrafish telencephalon. (A) Lesion and bromo-deoxyuridine (BrdU) treatment paradigm. Fish were treated with BrdU 3 days post lesion (dpl) for 6 hours before the sacrifice. (B) cxcr5 in situ hybridization on lesioned telencephalon. (B’) High magnification of the dorsomedial region in (B). (B”) High-magnification of the dorsolateral region in (B). (C) BrdU immunohistochemistry on lesioned telencephalon. (C’) High-magnification of the dorsomedial region in (C). (C”) High-magnification of the dorsolateral region in (C). (D) Merged image of (B) and (C). (D’) Merged image of (B’) and (C’). (D”) Merged image of (B”) and (C”). [file 1749-8104-7-27-S1.tiff]

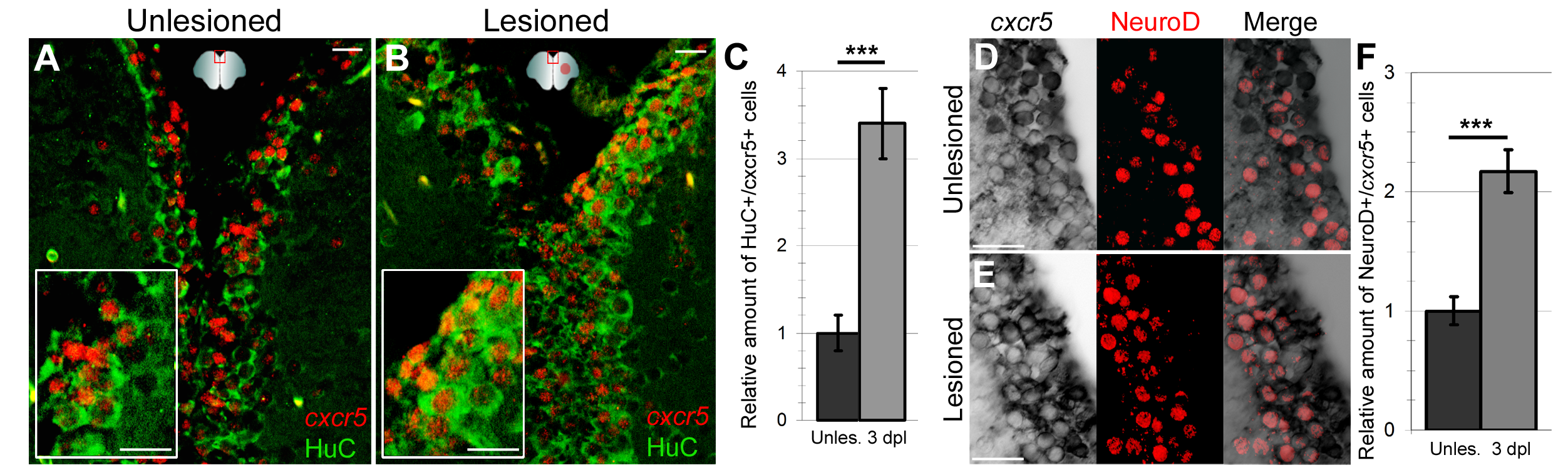

Supplement: Additional file 2 — Figure S2. Title: Neurons express cxcr5. (A) cxcr5 fluorescent in situ hybridization (FISH) coupled to HuC immunohistochemistry (IHC) on a section of unlesioned adult zebrafish telencephalon. Inset is magnified image. (B)cxcr5 FISH coupled to HuC IHC on a section of 3 days post lesion (dpl) adult zebrafish telencephalon. Inset is magnified image. cxcr5 is expressed in neurons before and after injury. (C) Quantification graph for cxcr5-expressing HuC-positive cells. (D)cxcr5 chromogenic in situ hybridization (ISH) coupled to NeuroD IHC in unlesioned telencephalon. NeuroD-positive differentiating neurons, which are several cell diameters away from the ventricle express cxcr5 (white asterisks). cxcr5 expression is weaker in NeuroD-positive neurons in comparison to cxcr5-positive cells closer to the ventricle (yellow asterisks). In a transition zone between strong NeuroD-positive (white asterisks) and NeuroD-negative cells (yellow asterisks), cells express NeuroD and cxcr5 weakly (blue asterisks). (E)cxcr5 chromogenic ISH coupled to NeuroD IHC in 3 dpl telencephalon. NeuroD-positive differentiating neurons are more numerous, dispersed inside the parenchyma distantly in comparison to unlesioned telencephalons and express cxcr5 (white asterisks). cxcr5 expression is weaker in NeuroD-positive neurons in comparison to cxcr5-positive cells closer to the ventricle (yellow asterisks). (F) Quantification graph for cxcr5-positive NeuroD-expressing cells. The number of NeuroD/cxcr5 double-positive cells increase upon injury in adult zebrafish telencephalon. Scale bars 25 μm; n = 4 telencephalons for every set of analyses. [file 1749-8104-7-27-S2.tiff]

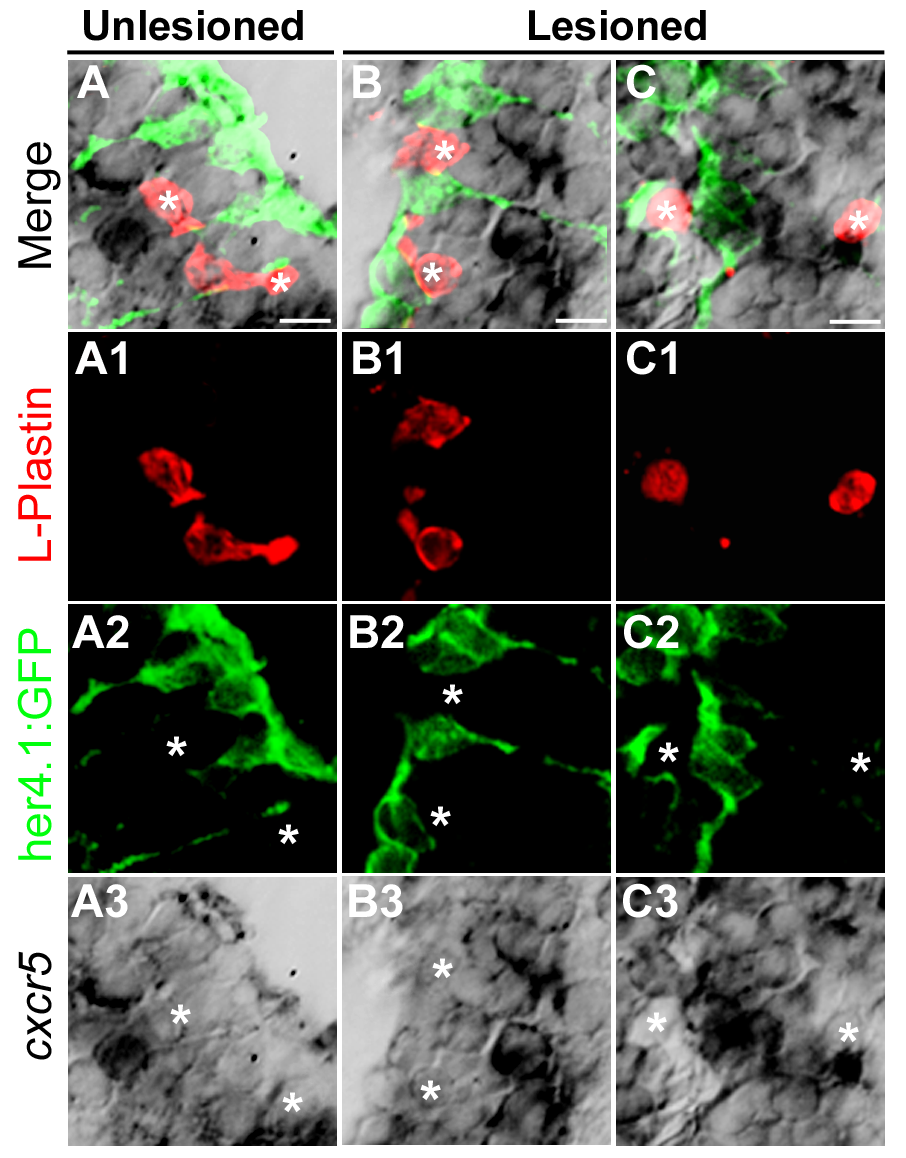

Supplement: Additional file 3 — Figure S3. Title: cxcr5 is not expressed in L-Plastin-positive cells in the adult zebrafish telencephalon. (A) cxcr5 in situ hybridization coupled to immunohistochemistry for L-Plastin (red, marking macrophages and microglia) and her4.1:green fluorescent protein (GFP) (green, marking the radial glial cells) in the unlesioned telencephalons. White asterisks indicate the L-Plastin cells. (A1-A3) Individual channels for L-Plastin, her4.1:GFP and cxcr5, respectively. (B) cxcr5 in situ hybridization coupled to immunohistochemistry for L-Plastin and her4.1:GFP in the lesioned telencephalons (dorsolateral region). White asterisks indicate the L-Plastin cells. (B1-B3) Individual channels for L-Plastin, her4.1:GFP and cxcr5, respectively. (C) cxcr5 in situ hybridization coupled to immunohistochemistry for L-Plastin and her4.1:GFP in the lesioned telencephalons (dorsomedial region). White asterisks indicate the L-Plastin cells. (C1-C3) Individual channels for L-Plastin, her4.1:GFP and cxcr5, respectively. [file 1749-8104-7-27-S3.tiff]

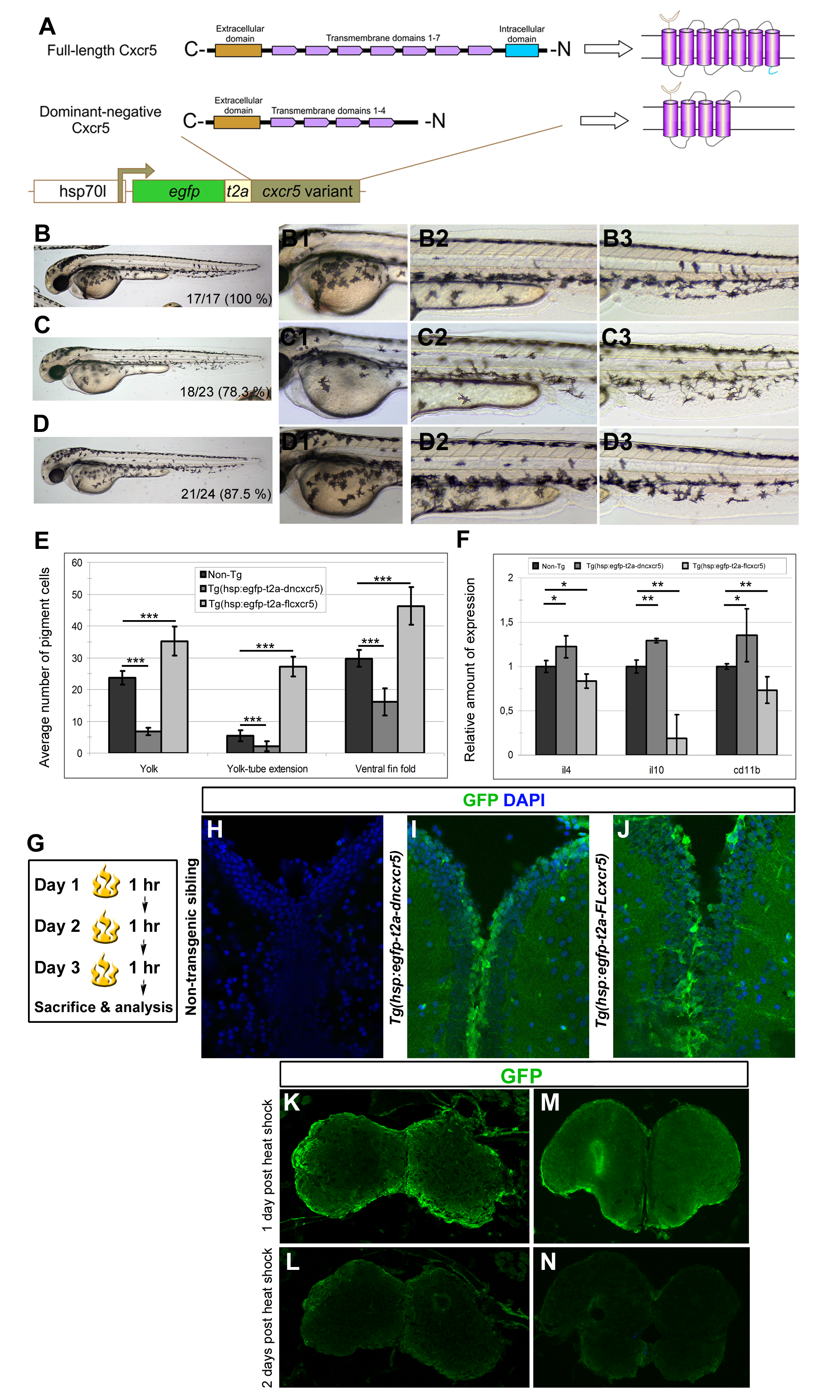

Supplement: Additional file 4 — Figure S4. Title: Larval phenotypes upon cxcr5 misexpression; and transgene activity in the adult zebrafish telencephalon. (A) Cxcr5 is a seven-span transmembrane protein with an extracellular receptor domain, seven transmembrane domains and a C-terminus intracellular domain. We generated full-length and dominant-negative versions of Cxcr5. Dominant negative variant lacks the last three transmembrane domains and the intracellular domain. Both variants are inserted into a transgenesis cassette that contains the coding sequence for enhanced green fluorescent protein (EGFP) and self-cleaving T2A peptide. The whole cassette is expressed under heat-inducible hsp70l promoter. (B) Non-transgenic sibling at 3 days post-fertilization (dpf). (B1) High-magnification image of the yolk-sac in B. (B2) High-magnification image of the yolk-tube extension in B. (B3) High-magnification image of the ventral fin fold in B. (C)Tg(hsp:egfp-T2A-dncxcr5) transgenic animals at 3 dpf after two heat shocks in gastrula. (C1) High-magnification image of the yolk-sac in C. (C2) High-magnification image of the yolk-tube extension in C. (C3) High-magnification image of the ventral fin fold in C. (D)Tg(hsp:egfp-T2A-FLcxcr5) transgenic animals at 3 dpf after two heat shocks in gastrula. (D1) High-magnification image of the yolk-sac in D. (D2) High-magnification image of the yolk-tube extension in D. (D3) High-magnification image of the ventral fin fold in D. (E) Quantification graph for the number of pigment cells in different regions of the non-transgenic, Tg(hsp:egfp-T2A-dncxcr5) and Tg(hsp:egfp-T2A-FLcxcr5) larvae. Note that dominant negative variant of cxcr5 significantly reduces while full-length cxcr5 significantly increases the number of pigment cells in comparison to the non-transgenic siblings. (F) Quantitative real-time PCR analyses at 3 dpf after the misexpression of cxcr5 with two heat shocks at gastrula. (G) Heat-shock scheme for adult zebrafish telencephalon expression. (H) GFP and DAP [file 1749-8104-7-27-S4.tiff]

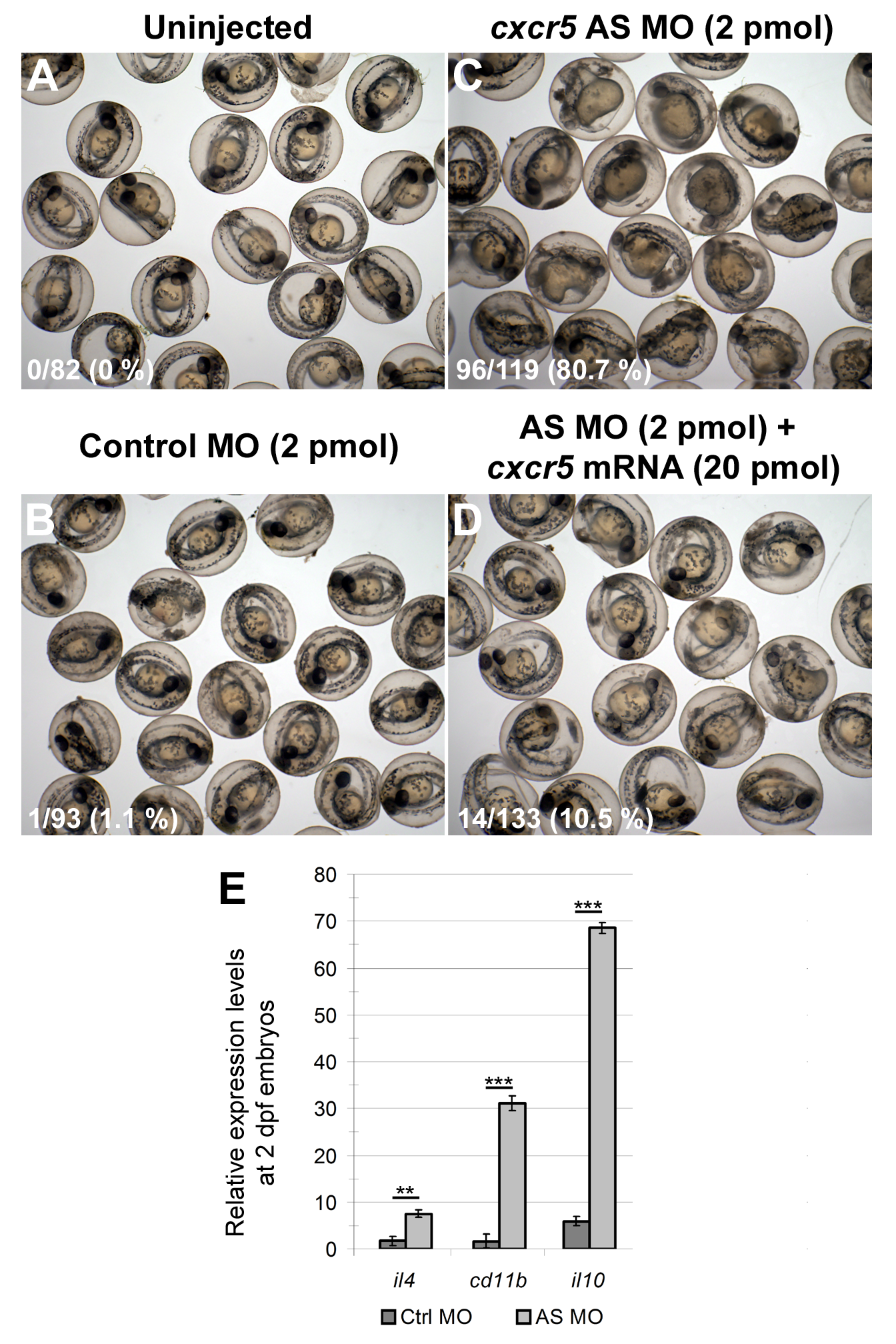

Supplement: Additional file 5 — Figure S5. Title: cxcr5 translation-blocking antisense morpholino is functional. (A) Uninjected 2 day post-fertilization (dpf) embryos. (B) Control morpholino-injected embryos show no morphological phenotypes at 2 dpf. (C)cxcr5 antisense morpholinoinjected embryos display severe anomalies in axial extension and head development. (D)cxcr5 mRNA rescues the knockdown phenotypes when co-injected with antisense morpholinos. (E) Quantitative real-time PCR analysis of genes regulated by cxcr5, upon control and antisense morpholino injections. All the genes tested are upregulated after knocking down cxcr5. Percentages in A-D represent the ratio of embryos with gross morphological defects to the whole clutch size. [file 1749-8104-7-27-S5.tiff]

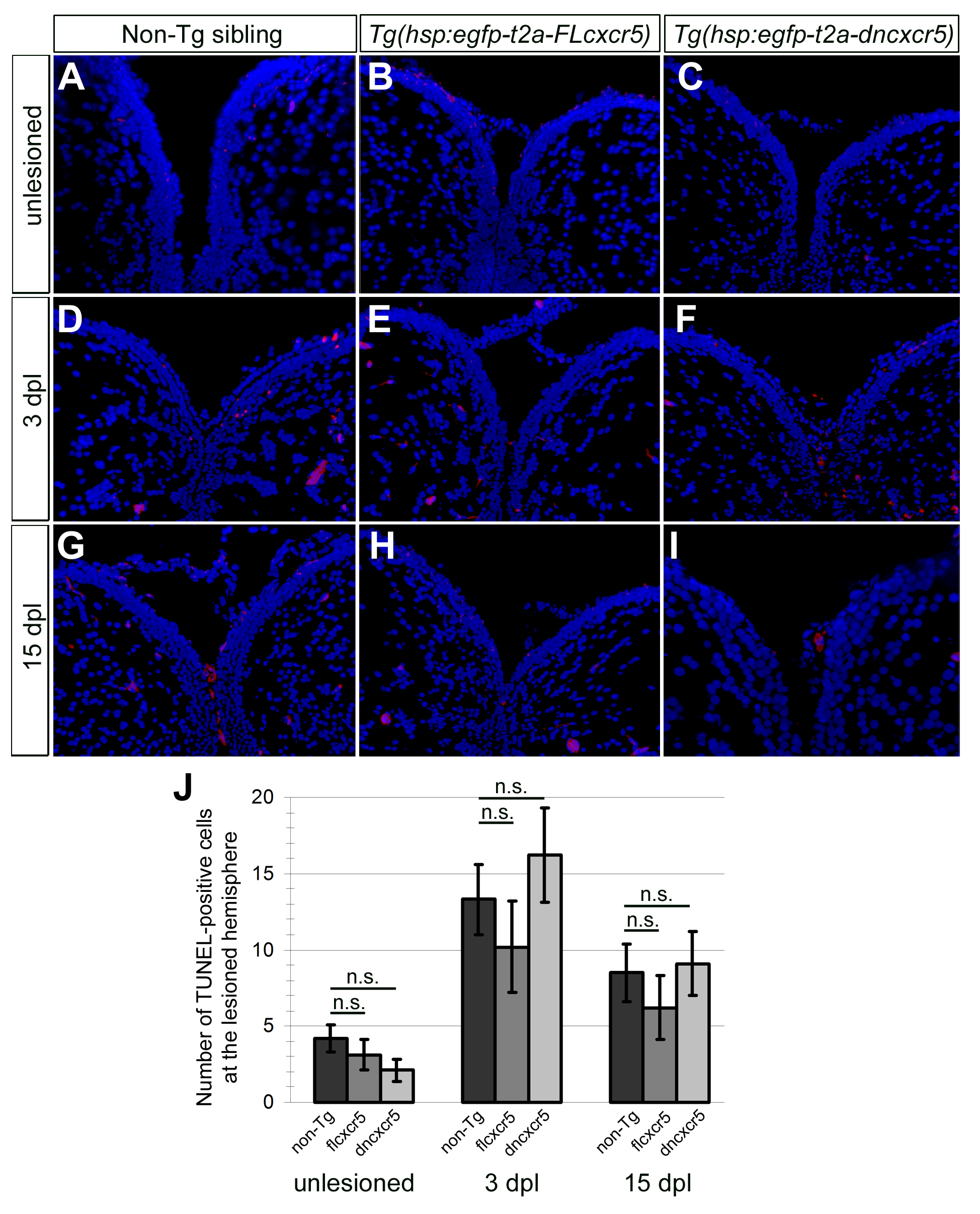

Supplement: Additional file 6 — Figure S6. Title: Misexpression of cxcr5 does not lead to cell death. (A-I) TUNEL staining to detect the apoptotic cells in non-transgenic siblings, Tg(hsp:egfpt2a-FLcxcr5) and Tg(hsp:egfp-t2a-dncxcr5) animals pre-lesion, 3 days post-lesion (dpl) and 15 dpl time points. Red nuclei indicate the apoptotic cells. (J) Quantification graph shows the number of apoptotic nuclei in the lesioned hemisphere. Misexpression of cxcr5 does not alter the levels of cell death. Two telencephalons were used for every time point. [file 1749-8104-7-27-S6.tiff]
