# Supplementary material for: The chemokine receptor cxcr5 regulates the regenerative neurogenesis response in the adult zebrafish brain
Source: Neural Dev. 2012 Jul 23;7:27. doi: 10.1186/1749-8104-7-27 (PMC3441421; doi:10.1186/1749-8104-7-27)
Supplement: Additional file 7 — Table S1. Title: qRT-PCR primers. [file 1749-8104-7-27-S7.doc]

**Supplementary Table 1: qRT-PCR primers**

| **Gene ID** | **Accession Number** | **Forward Primer (5’)** | **Reverse Primer (3’)** |
| --- | --- | --- | --- |
| *il4* | NM_001170740 | TTCCTGCTTGGCAGAGAGTT | GTGAATGGGATCCTGAATGG |
| *il10* | BC163038 | GCTCTGCTCACGCTTCTTCT | AAGGAAAGCCCTCCACAAAT |
| *cd11b* | XM_687072 | TCCTCGGATTCCAGAAACAC | GTCCTCCAATCACTCCTCCA |
